# Automated Classification of Normal Control and Early-Stage Dementia Based on Activities of Daily Living (ADL) Data Acquired from Smart Home Environment

**DOI:** 10.3390/ijerph182413235

**Published:** 2021-12-15

**Authors:** Lee-Nam Kwon, Dong-Hun Yang, Myung-Gwon Hwang, Soo-Jin Lim, Young-Kuk Kim, Jae-Gyum Kim, Kwang-Hee Cho, Hong-Woo Chun, Kun-Woo Park

**Affiliations:** 1 Convergence Research Center for Diagnosis, Treatment and Care System of Dementia, Korea Institute of Science and Technology, Seoul 02792, Korea; ynkwon@kisti.re.kr (L.-N.K.); sjlim@kist.re.kr (S.-J.L.); 2 Future Information Research Center, Korea Institute of Science and Technology Information, Seoul 02456, Korea; 3 Department of Computer Science and Engineering, Chungnam National University, Daejeon 34134, Korea; ykim@cnu.ac.kr; 4 Department of Data and HPC Science, University of Science and Technology, Daejeon 34113, Korea; yangdonghun3@kisti.re.kr (D.-H.Y.); mgh@kisti.re.kr (M.-G.H.); 5 Artificial Intelligence Technology Research Center, Korea Institute of Science and Technology Information, Daejeon 34141, Korea; 6 Department of Neurology, Korea University Anam Hospital, Korea University College of Medicine, Seoul 02841, Korea; fisher3721@naver.com; 7 Department of Biomedical Research Center, Korea University Anam Hospital, Seoul 02841, Korea; khacts@daum.net

**Keywords:** activities of daily living, aging population, early-stage dementia, instrumental ADL, machine learning, personalization

## Abstract

With the global trend toward an aging population, the increasing number of dementia patients and elderly living alone has emerged as a serious social issue in South Korea. The assessment of activities of daily living (ADL) is essential for diagnosing dementia. However, since the assessment is based on the ADL questionnaire, it relies on subjective judgment and lacks objectivity. Seven healthy seniors and six with early-stage dementia participated in the study to obtain ADL data. The derived ADL features were generated by smart home sensors. Statistical methods and machine learning techniques were employed to develop a model for auto-classifying the normal controls and early-stage dementia patients. The proposed approach verified the developed model as an objective ADL evaluation tool for the diagnosis of dementia. A random forest algorithm was used to compare a personalized model and a non-personalized model. The comparison result verified that the accuracy (91.20%) of the personalized model was higher than that (84.54%) of the non-personalized model. This indicates that the cognitive ability-based personalization showed encouraging performance in the classification of normal control and early-stage dementia and it is expected that the findings of this study will serve as important basic data for the objective diagnosis of dementia.

## 1. Introduction

Global demographic studies show that the proportion of the population aged 65 years and older increased to 9% of the total, which indicates the advent of an aging society. In the case of South Korea, the aging population is growing even faster, and the proportion of the population aged 65 years and older increased from 13.8% in 2017 to 16% in 2019 [1]. The trend toward an aging population entails problems such as increases in the number of patients with dementia, geriatric diseases, and the number of households with older adults living alone.

The number of dementia patients worldwide is estimated to be 50 million, and this is expected to increase to 82 million by 2030 and 152 million by 2050. Consequently, the global burden in terms of cost for dementia patient care has been estimated at $818 billion in 2015 and is expected to reach $2 trillion by 2030 [2]. In Korea, the proportion of the population aged 65 and over is expected to increase by 24.5% by 2030 and 38.1% by 2050, and this increase has already led to a sharp increase in the number of dementia patients with approximately 790,000 as of 2019. This is expected to increase to 1.36 million by 2030 and account for 10% of the total population of older adults [3].

Dementia imposes a considerable social and economic burden in terms of treatment cost, but there is a problem since there is no treatment or cure for the condition or means of altering the progression of dementia [2]. Medication prescribed for dementia only delays the progression of dementia and is effective only when the treatment begins at the early stage of dementia or at the mild cognitive impairment (MCI) stage [4]. Early detection of dementia is important because early diagnosis leads to early pharmacological and non-pharmacological interventions so that older adults can maintain independence in their daily lives for longer [5,6]. According to the US mild cognitive impairment (MCI) practice guideline, the importance of early diagnosis has been emphasized for changes in lifestyle, appropriate intervention, and future planning [7]. That is, early prediction and diagnosis of dementia can be seen as the key to dementia treatment under the current state of the treatments being developed.

Previous studies have shown that the rate of progression from MCI to dementia is 9.6% per year [8,9] and that older adults with MCI have a higher risk of dementia than the healthy elderly [7]. It is also more difficult for them to perform activities of daily living compared to the healthy group [10]. These reports indicate that understanding the importance of activities of daily living and their characteristics is instrumental in the early prediction of dementia.

Another problem with the aging population is the increase in the number of households with older adults living alone. In the United States, the proportion of households with a person aged 65 or older living alone amounts to 11.4% of the total number of households [11], and in Korea, the percentage of the total number of households is 7.5%, showing an annual increasing trend [12]. In Korea, lonely deaths of people aged 65 or older living alone account for 42% of the total cases of lonely deaths [13], posing a serious social problem.

In a clinical study [14] that followed up on the effects of living alone on the incidence of dementia, the elderly living alone had a 50% increased risk of dementia, and MCI patients living alone were diagnosed with dementia one year earlier compared to the elderly who were living with someone. In addition, it was reported that 21.6% of dementia patients living alone experienced an accident that resulted in physical injury or property damage during the study period [15], all of which highlight the importance of further research for early diagnosis of dementia in the elderly living alone.

To diagnose and identify the causes of dementia, comprehensive evaluations including medical history, assessment of cognitive function and mental state, physical examination and neurological examination, neuropsychological tests, assessment of activities of daily living, laboratory tests, and neuroimaging are required [16]. In particular, the diagnosis of dementia requires a cognitive function test (MMSE: Mini Mental State Examination) and neuropsychological tests (e.g., CERAD-K: the Consortium to Establish a Registry for Alzheimer’s Disease-K; CDR: clinical dementia rating) and assessment of the Activities of Daily Living (ADL). The ADL assessment must be performed for a dementia diagnosis, but since the assessment is performed through an ADL questionnaire with a patient or guardian, it is dependent on the subjective judgment of clinicians, which may lead to reduced accuracy.

Activities of daily living (ADL) can be classified in terms of abilities to engage in the more complex instrumental activities of daily living (instrumental ADL), including household chores, cooking, laundering, taking medications, shopping, managing finances, and using the telephone and public transportation [17], and the basic activities of daily living (Basic ADL), including bathing, dressing, eating, using the toilet, and ambulating [18]. In previous studies, instrumental ADL has been reported to show a decline in the early stage of dementia and even before this stage [19,20], and BADL was reported to decline more significantly in the later stages of dementia [21]. In addition, evidence confirms that when a person has difficulties with instrumental ADL, the condition progresses from a state of cognitive health to MCI [22,23], and difficulty with instrumental ADL was identified as an important factor in the progression from MCI to dementia [24].

In a previous study that assessed patients’ ADL through subjective interviews with patients or caregivers, the results demonstrated that there was a relationship between ADL and cognitive function for each type of dementia (four types) [19]. The difficulties in BADL reportedly increase for each stage as dementia progresses [25]. To improve the ADL assessment with a more objective approach, several studies have directly observed the subjects in a home environment or in a laboratory, quantified the ADL, and measured the different levels of ADL performance [26,27,28,29]. Studies that investigated the relationship between MCI and dementia based on ADL data collected through direct observation can be categorized into research that observed the daily lives of study subjects in a smart home environment and research that assigned ADL tasks to study the subjects’ performance in a laboratory environment with sensors and compared the results with those of the normal control group.

In a recent (2021) study conducted in a laboratory environment, MCI or dementia patients exhibited differences compared to healthy older adults with lower success rates in ADL tasks and more time taken for task completion. This demonstrated that objective assessment of ADL with IoT sensors was possible [30]. However, the method is limited since it can only be performed in a specific place during a specified time. Conversely, a smart home environment, which is an actual living space, has the advantage of overcoming the limitations of a laboratory environment by unobtrusively collecting ADL information in the space but is disadvantaged by requiring a longer observation period.

In related research, Dawadi et al. (2016) reported that a combination of machine learning and smart-home-based activity monitoring data (the Washington State University Center of Advanced Studies in Adaptive System (CASAS) dataset) enabled the assessment and status prediction of health and cognitive function in older adults. The study demonstrated a statistically significant correlation (*r* = 0.72) between the prediction of clinical assessment results using activity behavior (CAAB) and the cognitive score provided by a clinician [31]. In another study by Alberdi et al. (2018), for the detection of AD symptoms, a machine-learning-based predictive model was developed to map smart-home-based activity features to the health assessment score [32]. Moreover, the possibility of automated assessment of mobility, cognition (memory), and mood was confirmed using smart-home-based ADL data (CASAS dataset).

However, the data used in these earlier studies are limited in application since they do not take into account the special cultural lifestyles and weather of South Korea, the characteristics of living spaces, and the characteristics and circumstances of the elderly living alone in the early stages of dementia. In addition, since each person has different daily habits and patterns for instrumental ADL activities, it is necessary to establish individually different criteria for the average range of activities and activities beyond the average range. That is, since the criteria of the range of normal daily living are different for each individual, a personalized prediction must be made considering the activity duration and counts of the activities, which serve as the average and normal criteria of an individual instrumental ADL.

To study this, smart homes were assembled in the residences of elderly living-alone subjects (normal controls or early-stage dementia) in South Korea, and data for instrumental ADL were collected on detailed activities that are characteristic of early dementia patients. Statistical analysis techniques and machine learning algorithms were applied to the collected data and a prediction model was developed based on the personalized design and performance evaluation. This study can solve the problem that ADL assessment depends on the subjective judgment of clinicians. Furthermore, a personalized classification model can improve the performance. The term ADL indicates the instrumental ADL in the paper. The remainder of this paper is organized as follows. Section 2 describes the ADL-based smart home construction and related research methods. Section 2.1 introduces the smart-home-based activities of daily living and IoT sensors used in this experiment for ADL data collection, while also explaining smart home construction. In Section 2.2, feature selection, basic data preprocessing, and the feature generation methods are introduced. The classification models are introduced in Section 2.3 for the classification between normal controls and those with early-stage dementia. Section 3 describes the experiments, including the experimental environments and results. Lastly, Section 4 presents the conclusions of this work and describes the future research directions.

## 2. Method

### 2.1. Smart-Home-Based Activities of Daily Living

The assessment of daily living activities for the elderly, S-IADL (Seoul-Instrumental Activities of Daily Living) [33], as diagnosed by Korean clinicians, provides elements for status evaluations of early-stage dementia through diagnosis and ADL assessment. This has the advantage of considering the cultural lifestyle characteristics of South Korea during the assessment. The items of the S-IADL assessment included 15 different activities (using the telephone, shopping, preparing food/cooking, household chores, using transportation, walking outdoors, taking medications, managing finances, grooming, using household appliances, managing belongings, unlocking and closing entrance doors, keeping appointments, talking about recent events, and leisure activities or hobbies).

In this study, of the 15 S-IADL activities, six could be inferred using various sensors in the smart home environment to create objective indicators for assessment. In consultation with a clinician, cooking, household chores, taking medications, grooming, using household appliances, and unlocking and closing the entrance doors were selected as the inferred ADL assessment items. In addition, “indoor wandering” was also recorded as an activity, giving seven activities in total for the finalized selection for the assessment.

The ADL in the proposed approach is actually not the real ADL but the inferred ADL, because they are recognized by only sensors. However, the term ADL is used instead of the inferred ADL in the paper.

**Figure 1:**
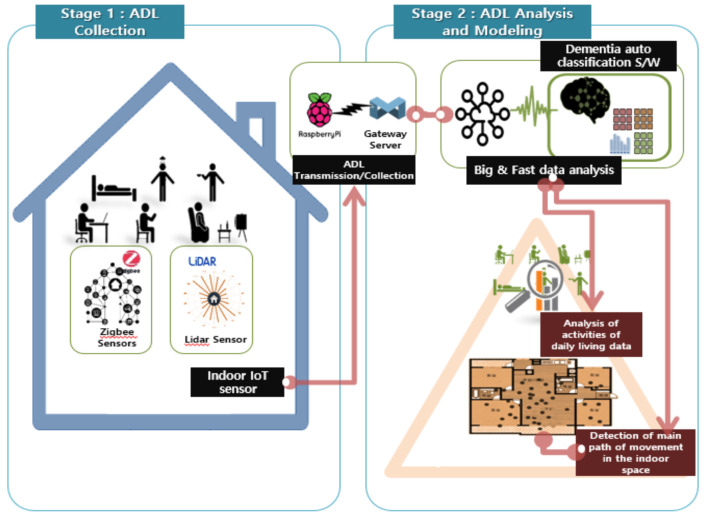
Study process for predicting ADL-based early dementia.

Figure 1 shows the flow chart for data collection and analysis of the present study. In Stage 1, ADL data were collected and transmitted using the IoT sensors installed in the actual living spaces of the study participants, and in Stage 2, the ADLs were analyzed and evaluated based on the collected data along with the indoor living patterns of the participants. Apart from the final stage of providing the analysis results to the users as a service, this paper describes the analysis and modeling of Stages 1 and 2.

**Figure 2:**
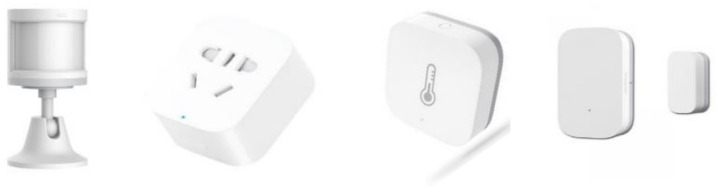
Examples of IoT sensors used in the experiment (motion sensor, smart plug, temperature–humidity sensor, door sensor).

To monitor and collect the data for the seven selected ADL activities, as shown in Figure 2 IoT sensors (door, motion, temperature–humidity, vibration, and lidar sensors and smart plugs) were installed on the household appliances that the study participants used in real life and various places inside their living spaces, as shown in Table 1 below:

The following functions are measured by the IoT sensors.

**Figure 3:**
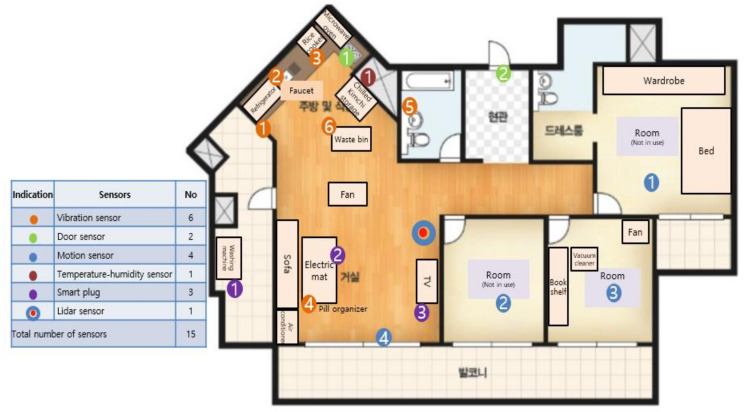
Floor plan of IoT sensor installation in the living space of a study participant.

The door sensors detect whether doors are open or closed and the time (in seconds) during which they are not completely closed. The vibration sensors detect vibrations and the inclination of each sensor is measured by its x, y, and z coordinates. The motion sensors detect a moving body and also the brightness (lux) values nearby. The temperature–humidity sensor measures the ambient temperature and humidity, and the smart plugs measure the length of time that electricity is used for home appliances. The Lidar sensor measures the x and y coordinates of a moving body to determine its trajectory and speed. Unlike a normal camera, the lidar sensor in this study did not risk breaching the study participants’ privacy and accurately recognizes a moving body even in the dark. Additionally, in preference to a wearable device, small commercial IoT sensors were discreetly installed in places that caused no impediments to indoor daily living. This reduced the psychological burden on the study participants, allowing them to live as normal. Figure 3 is a floor plan of the actual living space where a study participant resided and where the IoT sensors were installed.

The sensor data generated in real-time was temporarily stored using small Raspberry Pi and Zigbee Gateway PCs that are suitable for limited spaces and were unobtrusively installed in real living spaces. In addition, a separate and independent system was built using a portable LTE router to transmit data to the server. The required CPU and memory usage by the Raspberry Pi was for 80,000 daily records and 1.4 GB of data transmission per household while the Gateway system was designed for as many as 50 sensors to operate a smart-home-based data collection/transmission system.

In this study, the MOBIUS [34] IoT platform connected access points with various sensors and communication networks to other parts of the device to implement the smart home concept. The ADL sensing data were acquired by commercial IoT sensors and 2D-Lidar sensors using the Zigbee Gateway system. These data were then collected in the cube gateway through the MOBIUS Open Platform’s transport protocol (MQTT communication), and the raw data values were transmitted to the data analysis server (Mobius Server) through an internet network.

### 2.2. Feature Selection

#### 2.2.1. Basic Data Preprocessing

**Figure 4:**
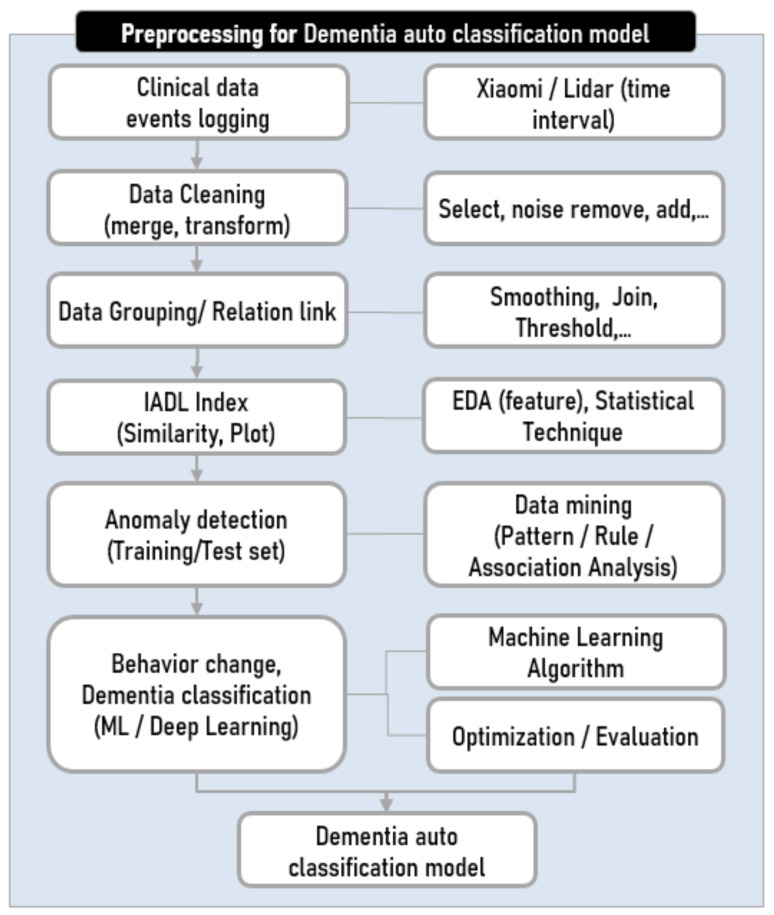
Dementia auto-classification model’s pre-processing flow chart.

As shown in Figure 4, the data were pre-processed for various analysis techniques to be applied to the raw ADL data that reflected the participants’ domestic living patterns. To understand the participants’ life patterns, the data collected from various IoT sensors and the Lidar sensor installed in the house were preprocessed for feature extraction. During pre-processing, information on the detection counts, changes recorded, or the duration of the detected movements and changes was collated per time unit and the ADL patterns were identified using the sensors’ combined records.

To detect the counts and duration of all motions indoors, a calculation (or conversion) was required for the raw data measured according to each sensor’s functionality.

To determine in which order, how often, and for how long the ‘pill organizer’, ‘kitchen sink faucet’, and ‘bathroom faucet’ were used, whenever the inclination (angle) of the ‘vibration sensor’ changed, it was counted as a single-use and the time until these appliances were returned to the original angle was calculated as the duration.

In the cases of taking a shower or face washing in a bathroom, or using a gas stove, there were changes in temperature and humidity, requiring a ‘temperature–humidity sensor’ to detect the change.

To identify the locations of movement, movement distance, and movement speed of a person in the living space, this information was calculated using the position values of the moving body collected from the 2D Lidar sensor.

In addition, because the time patterns of raw data detected from various sensors were different, the units were set to 10 min, one hour, or 1 day and the counts detected within the time unit and duration were time summed and calculated for each time unit.

Table 2 is an example of the data calculated by summing the status changes collected from the door sensor, motion sensor, smart plug, temperature–humidity sensor, and vibration sensor in a single day unit.

One ADL could be detected using one or a combination of multiple sensors. The sensor data to be combined for each type of ADL (taking medications, cooking, household chores, grooming, using household appliances, unlocking and closing entrance doors, and indoor wandering) were defined. The indoor wandering activity was measured by combining the values of the motion sensors and the coordinates collected from the lidar sensor.

Cooking activity refers to cooking rice or other dishes by preparing the ingredients in person and, according to its definition [33], this S-IADL item assesses the activity of cooking for oneself. Most Korean dishes have rice as a staple and rice cookers are used for cooking rice while a gas stove is used for cooking soup or side dishes. The ‘cooking’ activity in this study was defined as using two or more different kitchen appliances (e.g., rice cooker, refrigerator, microwave oven, kitchen sink faucet, gas stove, or cupboard) and the gas stove. In this case, the count of “cooking once” was based on the changed status values of the sensors (e.g., vibration sensors and door sensors) installed in kitchen household appliances and the temperature–humidity sensor on the gas stove indicating an increase in temperature. The time required for cooking (duration of cooking) was calculated using a combination of these values.

**Figure 5:**
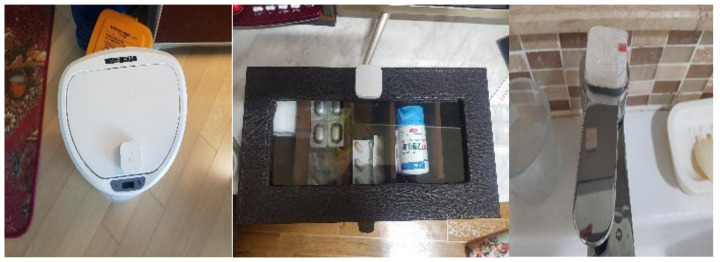
Examples of vibration sensor installation (bin, pill organizer, bathroom faucet).

According to the S-IADL assessment [33], the ‘taking medications’ activity item assesses “whether the study participant is taking the prescribed medication at a set time”. Rather than actually checking the behavior of taking medicine, it was decided that this activity was assessed by checking whether the participant used the pill organizer at the set time. To measure the count of taking medication activity, a separate pill organizer was specially made for this study, and a vibration sensor was attached to the lid in Figure 5. When the vibration sensor detected the change in inclination (angle), a single count of taking medication was recorded and the time required to return it to the original inclination was calculated as the duration for taking medication.

The ‘using household appliances’ activity item in the S-IADL assessment [33] checks whether the study participant can turn on/off the household appliances and use the appliances by manipulating buttons. By using a smart plug, whether the TV and electric mat were used once was determined when the electricity usage changed and, for other household appliances (e.g., the fan or vacuum cleaner), their use status was determined based on the vibration sensors’ detection counts.

The ‘unlocking and closing entrance door’ activity item of the S-IADL assessment [33] checks whether the participant opened and closed the door correctly using a key or password. Because this is an item that is difficult to assess accurately without a camera, we replaced the assessment item with a similar behavior that is exhibited when going out, which is equivalent to unlocking and closing door activity, in consultation with a clinician. Patients with dementia often forget to turn off power for household appliances when they go out. Accordingly, it was determined that when a participant turned off household appliances such as the TV or electric mat and the light in the living room when going out, it was counted as a single closing of the entrance door. To this end, the exit status was detected using the door sensor and the living room motion sensor while the locking/closing-the-door and the duration-of-being-out counts were calculated using the combination of changes in the electricity usage detected by the smart plugs and lux value of the indoor lighting.

The ‘household chores’ activity item of the S-IADL assessment [33] checks whether the participant performs household chores such as cleaning, washing dishes, home maintenance and repair, sweeping around the house, and hand washing clothes. We defined the count of ‘household chores’ activity as how often ‘washing dishes’ and ‘laundering’ were performed by the participant. ‘Washing dishes’ was defined as the activity of using the kitchen sink faucet, and the activity status was calculated using the vibration sensor installed in the kitchen sink faucet.

The ‘laundering’ activity was defined as the activity of using a washing machine, and the calculation was based on the electricity usage of the smart plug installed on the washing machine. Notably, older adults living alone do not tend to have many pieces of laundry and often wash their clothes by hand, but since this study did not use a camera for data collection, this activity could not be measured.

The ‘grooming’ activity item of the S-IADL assessment [33] checks ‘whether a participant performs grooming activities such as combing hair, shaving, makeup, and nail clipping by themselves’. We installed vibration sensors in the showerhead faucet, bathroom washbasin faucet, and hairdryer to detect whether the participants performed ‘grooming’ activities such as showering, hand washing, and hair styling by themselves.

The ‘indoor wandering’ activity item detects abnormal behavior patterns such as wandering around many different areas in the house for a specific time or staying in a place such as the porch area for a long time without specific reasons.

For example, staying in front of a TV for more than an hour at night (after 11 pm) is not an abnormal behavior; however, if the place is the area around the front door, this can be viewed as an ‘indoor wandering’ activity. For this purpose, the main location of movement and gait speed of the participant were identified using the ‘2D Lidar’ sensor, which can detect changes even in darkness. The time spent during one visit was analyzed to identify indoor living patterns. As shown in Figure 3, the indoor space was zoned (zones 1 to 6) for each household, and for each zone, the number of visits to the zone and the durations of stay for the visits were analyzed to determine the participants’ indoor life patterns.

**Figure 6:**
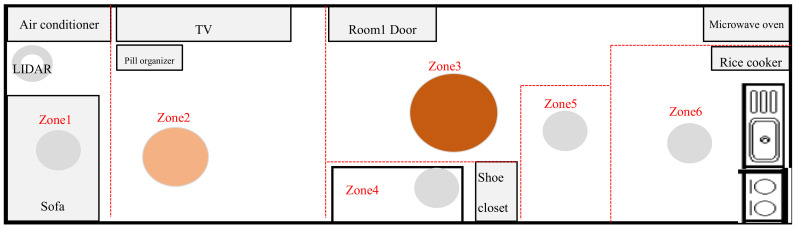
Example of wandering analysis for each indoor zone (Zone 1–Zone 6) in the living space of participant ’A’.

In Figure 6, the areas indicated by large circles and darker colors are the areas where the participant stayed the most. In the case of participant ‘A’, Zone 3 in front of the entrance was identified as the location with the longest duration of stay, followed by Zone 2 in front of the TV. For other zones, such as Zone 1 and Zone 4–Zone 6, the participant stayed for a similar duration. The hourly pattern of usual indoor wandering of participant ‘A’ was hereby identified. Through this analysis, the average number of visits per zone and average duration of stay for each zone were identified to analyze changes in the daily or hourly patterns of the participants.

#### 2.2.2. Personalization

Even for healthy participants, individual daily life had various patterns for each ADL activity depending on personal preferences in food and lifestyle with each individual having different criteria to define the range of normal daily life. For example, if the participant had a preference for bread or snacks, there was a significant difference in the number of, and average, cooking times compared to the participant who preferred rice-based meals. Since there was a difference in activity duration or frequency corresponding to the normal range for each individual, personalized prediction that took into account the individual’s average activity patterns was required rather than the determination of abnormal behavior based on uniform criteria.

Therefore, the criteria for ‘anomaly detection’ were applied with individual personalization that considered the average ADL activity pattern and cognitive ability of each individual for personalized dementia/normal classification.

In cases of normal participants with high cognitive abilities (MMSE), the anomaly detection reference ranges were broadly set, and in cases of low cognitive ability, the anomaly detection ranges were reduced. Therefore, even when the same activity was performed, stricter criteria for anomaly detection were applied to the activities of persons with dementia. This was to establish a difference between the activities of a normal control and a person with dementia in consideration of their different cognitive abilities. Considering that persons with dementia make more mistakes than persons without dementia, the criteria were set for more frequent responses to improve the prediction performance.

To this end, every participant was assigned into one of four groups: three normal control groups and one early-stage dementia group (Table 3). A range of anomaly detection criteria was set with different upper and lower limits based on the IQR (interquartile range), and the counts of activities beyond the average and normal activity ranges of ADL were calculated for each level of individual cognitive function (MMSE score), with this being used as the feature for analysis.

#### 2.2.3. Feature Generation

In this study, the data for analysis can generally be categorized as ‘IoT sensor data’ and ‘ADL data’ (Table 4).

The ‘IoT sensor data’ include the number of movements (detection) over a specific time (IoT Count) collected by the IoT sensors installed in many different places in the house and the duration for each detection period (IoT Duration), changes in temperature and humidity, and the movement distance and gait speed information calculated from the location information of the participant collected from the Lidar sensor.

The ‘ADL data’ are the previously selected ‘indoor wandering activity’ and six different ADL activities. This information was collected using a combination of multiple sensors. The ADL data can be divided into the number of times a specific ADL was performed, the ADL count data, the time required to perform each ADL, and the ADL duration data.

As shown in Table 5, the ADL feature sets created for each item of ADL activity are outlined as follows:

For the ‘taking medications’ activity, as well as the information on the taking-medication counts collected from the sensor, the time periods were divided (morning, lunch, dinner, and night), and features were generated to check whether the participant had regularly taken medications in comparison with the usual life patterns.

For the ‘cooking activity’, as well as the information on the cooking activity counts, the periods for eating were divided (morning, lunch, dinner, and night), and whether the participant cooked at the corresponding period were generated as features. Another feature was generated for cases when the duration of cooking was longer than 30 min. In addition, the result of analyzing the correlation between the appliances used for the normal controls and the early-stage-of-dementia group using the applied statistical techniques (see Section 3.2 for details) was reflected in the feature generation. That is, the number of simultaneous uses of highly correlated kitchen appliances used simultaneously during cooking, ‘refrigerator—kitchen sink faucet’, ‘kitchen sink faucet—rice cooker’, ‘refrigerator—gas stove’, and ‘gas stove—microwave oven’, were generated as features.

For the ‘grooming’ activity, it was important to identify the frequency and duration of all usages of the showerhead faucet, washbasin faucet, and hairdryer in the bathroom during all 24 h of the day, but in particular, features were generated by dividing the time of use into nighttime and daytime.

For the ‘using household appliances’ activity, features were generated by dividing the use of the TV and electric mat by time period (morning/late-night hours, daytime/nighttime, all day). In addition, the time spent watching TV within 30 min of returning home from going out was checked to determine how dependent the participant was on TV, and this was added as a feature.

For the ‘unlocking and closing entrance door’ activity, if household appliances (e.g., TV and electric mat) were detected to remain on after going out, this was regarded as not locking/closing the entrance door properly, and these occurrences were added as a feature.

For the ‘household chores’ activity, data of action of using the washing machine and data of action of using the sink faucet for 30 s or longer in the kitchen were generated as a feature.

For the ‘indoor wandering’ activity, the number of visits and the duration of stay for each zone (Zone 1 to Zone 6) of the indoor space set for each household was analyzed, and the frequencies of movements to the living room and other rooms were added as a feature. In particular, the number of times all data changes were detected at night (00:00–05:00) and the wandering activity in the indoor space at nighttime were added as a feature.

### 2.3. Classification between the Normal Controls and Those with Early-Stage Dementia

#### 2.3.1. Statistical Method-Based Classification

Statistical analysis and machine learning techniques were applied to the automated classification of the normal controls and those with early dementia.

Statistical analysis was used to determine which subcategories of the ADL activities showed significant differences between the normal controls and the early-stage dementia group and examine the differences in appliance-use between the two groups. The first condition for analyzing the difference between the two groups was to confirm whether the population followed a normal distribution. For this purpose, the normality of the population was determined using the Shapiro–Wilk test [35]. If the distribution was not normal, the Wilcoxon rank-sum test [36], a non-parametric test method, was used to compare the groups. To investigate the differences in the correlation between the appliances used during ADL-subcategory activities between the normal controls and early-stage dementia group, the normality of the population was tested first, and if the distribution was not normal, Spearman correlation analysis, a non-parametric test, was performed.

The Spearman correlation analysis is a nonparametric, ranking, statistical method; it was proposed by Charles Spearman in 1904. It measures the degree of association between two variables without any assumptions regarding the frequency distribution of the underlying variables. Spearman correlation analysis derives a correlation coefficient (r) by ranking data in order from small to large values, and the analysis determines whether there is a correlation between two variables. It is a method for calculating the correlation coefficient using the rank of two values, instead of the actual values of the two datapoints for which the correlation coefficient is to be calculated. The method is useful when there is an outlier in the data or when sample sizes are small.

The range of values is [−1, 1]; here, 1 indicates that the rank of one variable increases with the rank of the other, whereas −1 indicates that the rank of one variable decreases as the rank of the other increases. Furthermore, 0 suggests that the rank increase on one side is not related to the rank on the other side [37].

This is formula of the Spearman correlation coefficient:

(1) $$r=1-\frac{6\sum d_{i}{}^{2}}{n\left(n^{2}-1\right)}$$

where *d_i_* is the difference between the *x_i_* ranking (ranking when *x_i_* is listed in the ascending order from smallest to largest) and the *y_i_* ranking.

#### 2.3.2. Machine-Learning-Based Classification

**Figure 7:**
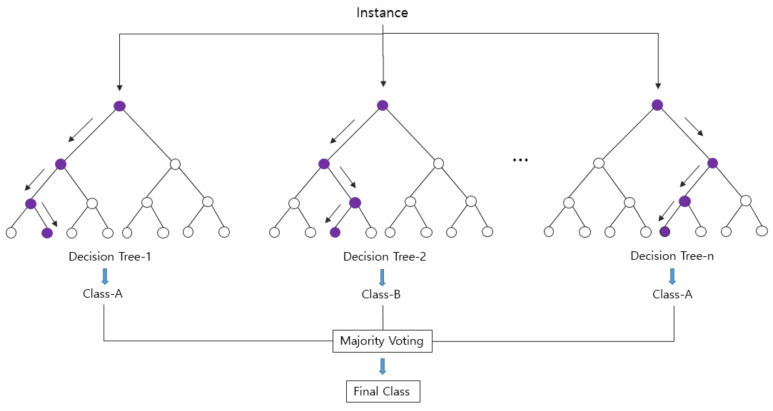
Classification process based on the RF algorithm.

Machine learning (ML) techniques are an essential area of research in the fourth industrial revolution, and ML-based approaches have demonstrated remarkable performance in various fields [38,39,40,41,42]. In this study, a model for classifying normal controls and the early-stage dementia patients was developed using the random forest (RF) method, which is reported to have excellent classification performance. RF is a type of ensemble learning method for classification and regression, and its basic architecture is shown in Figure 7.

RF is a classification technique that was developed by Breiman (2001); its primary advantage is that the designer of the model is free from the selection of the input variables. Therefore, it is suitable for fields requiring classification or prediction from hundreds of independent variables and vast amounts of learning data [43].

**Figure 8:**
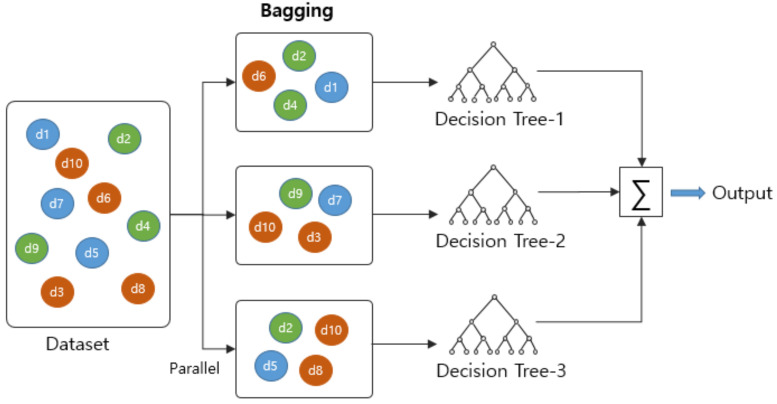
Bagging and RF process.

For a given dataset, after random separation using the bagging method, an ensemble technique and multiple trees are generated with the respective separated datasets Figure 8. The final predicted classification value is derived by applying the majority rule to the output value of each generated tree. Although the accuracy of each tree may be low, by using multiple trees, the method has high prediction accuracy and learning stability [44].

## 3. Experiments

### 3.1. Experimental Environments

Since ADL showed a decrease from the early stage of dementia or even before, for the early detection of such changes in ADL, the participants in this clinical study were divided into an early-stage dementia patient group and a normal control group. The criteria for recruitment and inclusion were that these participants were elderly and living alone. Second, in the case of dementia patients, those with a legal representative were recruited based on the National Institute of Toxicological Research ethical standards for clinical studies, which stipulate that dementia patients may only participate in a clinical study with the consent of their legal representatives. Third, the clinical dementia rating (CDR) score of patients with early-stage dementia criterion was set at 0.5 to 1 point. The interviews of cognitively healthy elderly and early-stage dementia candidate participants consisted of an MMSE, CDR test, and ADL assessment, as shown in Table 6. In total, 13 elderly individuals living alone were recruited, seven normal controls and six with early-stage dementia, of whom 12 were women and 1 was male, aged on average 78 years or older. The study participants were recruited in cooperation with the Dementia Care Center in Seoul, and the normal controls and patients with early-stage dementia were selected after MMSE, CDR, and ADL tests performed by a clinician at a university hospital.

The data used for analysis were regularly collected over approximately 13 months from May 2020 to June 2021, during which time data for one week or two weeks were collected every month for each participant. The data collection period was different for each participant. Of the 13 participants in the clinical study, the average age of the normal controls was 78.8 years, and the average age of patients with early-stage dementia was 80.3 years. In terms of the average cognitive function score of the normal control group, the MMSE score was 27.5 points and the CDR score was 0 points. For participants with early-stage dementia, the average MMSE score was 17.6, and the average CDR score was 0.92. By the end of the clinical study, there was a change in the scores in the MMSE test results of seven participants in the normal control group. There were slight changes such as an increase in the MMSE score (two participants), no change in the MMSE score (one participant), and a decrease in the MMSE score (three participants), but the overall MMSE score of the group was the same at 27.5 points, and the CDR score remained unchanged at 0.

The data for statistical analysis were used to determine the difference in ADL activities and utilized instruments between the normal controls and early-stage dementia group, and the data were divided into two categories, ‘normal’ and ‘dementia’.

Data were extracted to analyze the difference between ADL activities, the duration data for each ADL (e.g., grooming, cooking, household chores, and household appliances) corresponding to the ADL activities’ data, and the count data for the number of times all instruments were utilized. The collected data were divided into two groups (Normal and dementia) and statistical techniques (Shapiro–Wilk test, Wilcoxon rank-sum test, etc.) were applied for analysis.

To analyze whether there was a difference in the movement distance and gait speed between the two groups, the relevant data collected from the lidar sensor were extracted and divided into two groups (normal and dementia), and statistical techniques were applied.

For analysis of the differences and correlations between the utilized instruments, the count data collected from the sensors were extracted, divided into the control and early-stage dementia groups, and statistical techniques (Shapiro–Wilk test and Spearman’s correlation) were applied.

**Figure 9:**
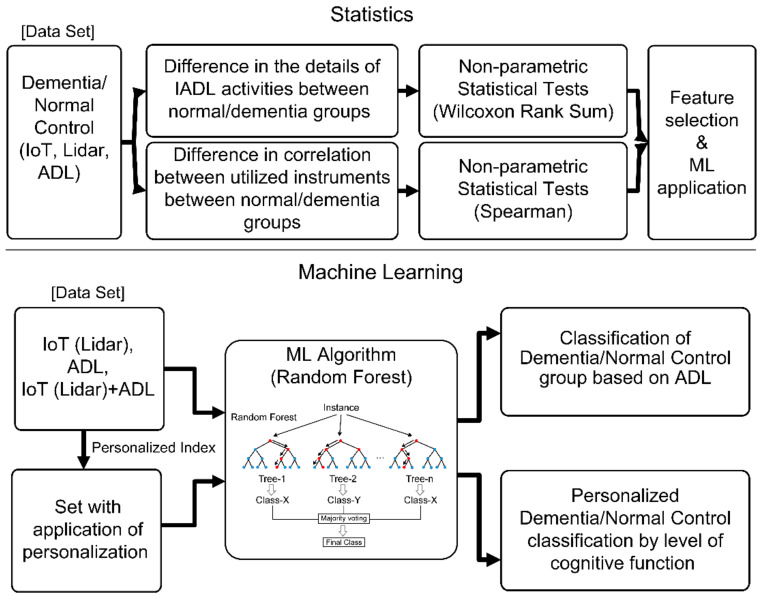
Data analysis method.

For training of the normal controls’ dementia auto-classification model, the feature sets were composed as shown in Figure 9, and supervised learning was performed for the classification of normal controls and dementia groups.

In Figure 9, the IoT dataset, including the Lidar sensor data, is designated ‘IoT’, the dataset for the ADL data is designated ‘ADL’, and the total dataset combining both IoT and ADL data is designated ‘IoT + ADL’. The training was then performed by dividing the datasets with the application of personalization, and performance assessment was performed for each model. For all training data (IoT, Lidar, and ADL), data calculated in the time unit of one hour were used.

Personalization is the application of different criteria according to an individual’s cognitive ability. In other words, even in normal people, if the cognitive ability is somewhat low, the standard of low abnormal symptom detection is applied.

The ‘dataset applied with personalization’ is a dataset that differentiates all the data by individual cognitive ability (MMSE) and calculates the number of times that the value is beyond the range of the average and normal activity criteria (anomaly detection) (see Section 2.2.2).

When modeling, data from the normal and early-stage dementia groups were mixed; however, the data contain a unique id for each person. Accordingly, previous ADL records were analyzed to determine the individual standards and whether new the ADL instances were abnormal.

### 3.2. Experimental Results

The control and the early-stage dementia groups’ use count data were analyzed to determine whether there was a statistically significant difference between them for subcategories of ADL activities performed using household appliances. The count data of the sensors installed in places common to all study participants, such as entrances, microwave ovens, gas stoves, TVs, washing machines, pill organizers, refrigerators, rice cookers, kitchen sink faucets, and bathroom faucets, were extracted for analysis. The normality of the extracted data groups was tested using the Shapiro–Wilk test, but since none of the data groups followed a normal distribution, the non-parametric Wilcoxon test was performed. As shown in Table 7, the analysis results show no statistical difference between the normal controls and the early-stage dementia group in terms of the counts for ‘microwave oven’ and ‘pill organizer’, but all other appliances (entrance, gas stove, TV, washing machine, refrigerator, rice cooker, kitchen sink faucet, and bathroom faucet) showed a statistically significant count difference with very small *p*-values below the significance level (α) of 0.05.

Next, we analyzed whether there was a statistically significant difference between the two groups regarding the duration data for ADL activities. The ADL activities include household chores, cooking, grooming, using household appliances, taking medications, and unlocking and closing the entrance doors. Here, ‘taking medications’ was excluded because it was analyzed by counting the uses of the pill organizer. For the ‘using household appliances’ activity, the duration of TV, electric mat, and fan use was analyzed, and for the ‘household chores’ activity, the duration of washing dishes and laundering was extracted for analysis. The normality of the extracted data groups was tested using the Shapiro–Wilk test but since the data group did not follow the normal distribution, the non-parametric Wilcoxon test was performed. As shown in Table 8, the analysis of the results for the duration of all ADL activities (e.g., grooming, using household appliances, and cooking) other than household chores (washing dishes and washing machine), had a *p*-value of less than 0.05, with a significance level (α) of 0.05, indicating a statistically significant difference between the normal controls and the early-stage dementia group.

In addition, we analyzed whether there was a difference in the correlation between the household appliances used by the normal controls and the early-stage dementia group. For these fittings, appliances, and furnishings, the count data of the entrance, microwave oven, TV, washing machine, gas stove, pill organizer, refrigerator, rice cooker, kitchen sink faucet, and bathroom faucet were extracted for both groups. Since the extracted data did not follow a normal distribution, Spearman correlation analysis was performed.

**Figure 10:**
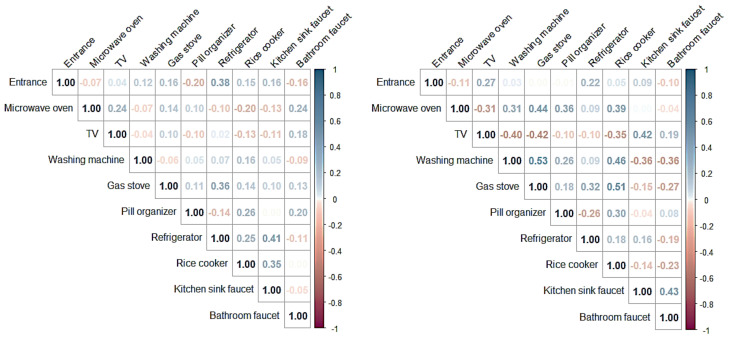
Correlation analysis between appliances used for normal controls (**left**) and early-stage dementia groups (**right**).

As shown in Figure 10, the analysis results showed the correlations between appliances in the normal control group to be *r* = 0.41 for ‘kitchen sink faucet—refrigerator’, *r* = 0.38 for ‘refrigerator—entrance’, *r* = 0.36 for ‘refrigerator—gas stove’, and *r* = 0.35 for ‘kitchen sink faucet—rice cooker’. These results confirmed that the normal control group mainly showed a correlation between the appliances related to cooking.

Conversely, in the early-stage dementia group, the correlation between appliances was *r* = 0.53 for ‘washing machine—gas stove’, *r* = 0.51 for ‘rice cooker—gas stove’, *r* = 0.46 for ‘rice cooker—washing machine’, *r* = 0.44 for ‘gas stove—microwave oven’, *r* = 0.43 for ‘kitchen sink faucet—bathroom faucet’, and *r* = 0.42 for ‘kitchen sink faucet—TV’. Notwithstanding the correlation between ‘rice cooker—gas stove’, which is directly related to cooking, the correlations between the other appliances were difficult to interpret. That is, the results show that it is difficult to identify patterns of using appliances that are related to each other in the daily life of early-stage dementia patients compared to the cases of normal controls.

**Figure 11:**
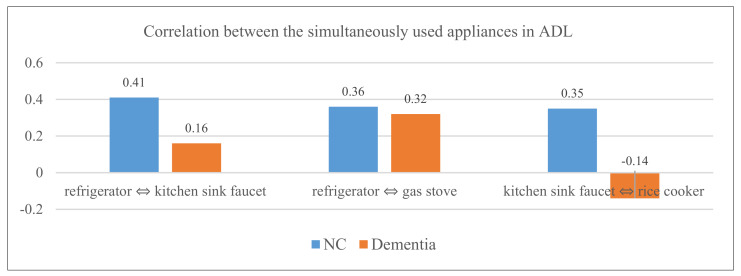
Difference in the correlation between the simultaneously used appliances in the normal control group and the early-stage dementia group.

As shown in Figure 11, the appliances used simultaneously that showed differences in the correlation between the normal control group and the early-stage dementia group are as follows:

- ‘refrigerator ⇔ kitchen sink faucet’: normal control group (*r* = 0.41), early-stage dementia group (*r* = 0.16);
- ‘refrigerator ⇔ gas stove’: normal control group (*r* = 0.36), early-stage dementia group (*r* = 0.32);
- ‘kitchen sink faucet ⇔ rice cooker’: normal control group (*r* = 0.35), early-stage dementia group (*r* = −0.14).

The results obtained from analyzing correlations between appliances used during ‘cooking’ activities (e.g., kitchen sink faucet—refrigerator, refrigerator—gas stove, kitchen sink faucet—rice cooker, and rice cooker—gas stove) significantly contributed to creating new features which were identified as important in the development of the ADL-based dementia auto-classification model.

Next, we analyzed whether there was a statistically significant difference between the normal control group and the early-stage dementia group in terms of the indoor movement distances and gait speeds. The indoor movement distances were measured by collecting the distance moved by time period through the 2D Lidar sensor, and the gait speed was calculated in kilometers per hour (km/h). The normality of the data group was tested, but the data distribution did not follow a normal distribution; therefore, the non-parametric Wilcoxon rank-sum test was used. The analysis showed that there was no significant difference (*p*-value = 0.3228) in the indoor movement distances between the normal controls and early-stage dementia group while, the average gait speeds in the normal control group (10,584 cases) and the early-stage dementia group (6408 cases) were 1.023 km/h and 1.209 km/h, respectively. As shown in Table 9, the *p*-value was very small (*p*-value < 2.2 × 10^−16^) at a significance level (α) of 0.05; there was a statistically significant difference between the two groups.

In this study, RF was used as the machine learning algorithm for modeling normal controls–early-stage dementia auto-classification. The feature sets used for training were divided into IoT, ADL, and IoT + ADL for comparative analysis. The effect of personalization was analyzed by separating the data with personalization from the data without personalization.

As shown in Table 10, the number of features generated from each IoT dataset was 65 for IoT_Duration, 65 for IoT_Count, and 2 for Lidar, giving a total number of 132 IoT features. The number of features generated from the ADL dataset was 31 for ADL_Duration and 32 for ADL_Count, totaling 63 ADL features. The number of features generated in this study was 195.

The normal controls–early-stage dementia classification model was evaluated and validated using 10-fold cross-validation.

**Figure 12:**
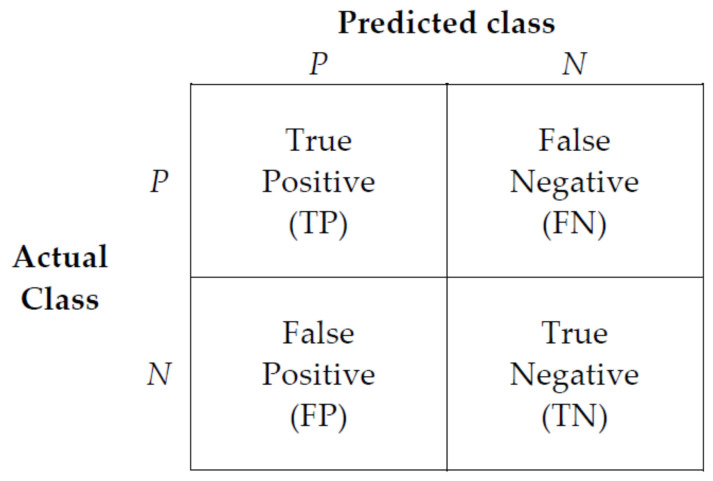
Confusion matrix.

When evaluating a classification model, the accuracy of the classification results of the model must be evaluated, and the confusion matrix in Figure 12 is a summary of the prediction results for a classification problem.

- True Positive (TP): Correct prediction as true when the actual class is true (correct prediction);
- False Positive (FP): Incorrect prediction as true when the actual class is false (incorrect prediction);
- False Negative (FN): Incorrect prediction as false when the actual class is true (incorrect prediction);
- True Negative (TN): Correct prediction as false when the actual class is false (correct prediction).

Precision, recall, *F*1-*score*, and accuracy were used as indicators to evaluate the performance of the classification model. Precision (Equation (2)) refers to the predicted and actual data ratio values both being positive among the classes with “positive” predicted values. Recall (Equation (3)) indicates that the predicted and actual data ratio values are both positive among the classes with “positive” actual values. *F*1-*score* (Equation (4)) is the harmonic mean of precision and recall, and the accuracy (Equation (5)) indicates the degree of correct classification between normal controls and patients with early-stage dementia.

(2) $$Precision=\frac{TP}{TP+FP}\;$$

(3) $$Recall=\frac{TP}{TP+FN}\;$$

(4) $$F1-score=2\times \frac{Precision\times Recall}{Precision+Recall}$$

(5) $$Accuracy=\frac{TP+TN}{TP+TN+FP+FN}$$

Table 11 shows that in the case of the IoT + ADL (all data) feature model, the best classification accuracy of 91.2% could be achieved when the RF algorithm was used in the model with personalization.

As RF is known to be suitable for performing classification from a large number of independent variables, in this study, using a total of 195 features (IoT + ADL), high performance was achieved.

Furthermore, the model performance after personalization (91.20% accuracy) was superior to that before personalization (84.54% accuracy).

It is noteworthy that the IoT performance (86.80% accuracy) was superior to the ADL performance (83.47% accuracy). The significance of the implication is that although ADL was possibly under-represented by the used sensors, rather than using features that are limited to specific activities identified by dementia experts, big data obtained from various sensors, which more broadly reflect the aspects of daily living, showed superior performance.

SHAPley additive exPlanations (SHAP) was used to determine the importance of features in predictive models. SHAP is an algorithm based on the Shapley game theory values to explain the output of machine learning models. Shapley values are obtained by combining multiple variables to determine the importance of a specific variable and derive the average change according to the presence or absence of a specific variable [45].

The importance of features was calculated using SHAP, and the contribution of each feature to the overall result was numberized. The contribution of each feature could be expressed as a change in the overall result when the contribution of a specific feature was excluded.

The following Table 12 shows the importance ranking of features in the IoT + ADL feature model, which had the highest performance among the machine learning models used experimentally in this study.

From examining the importance of features after personalization, the late-night life patterns were identified as important. Contrary to the expectation that daytime data would have the larger impact because of the availability of much daytime data, the late-night behavior contributed significantly to the classification performance.

## 4. Conclusions

This study developed a smart home ADL-based normal controls and early-stage dementia subject classification system. To this end, this study established smart home environments applying both IoT sensors and 2D LIDAR sensors in actual residential areas to obtain objective ADL data. The main contributions of this study are as follows.

First, this study verified that the classification model developed in this study can be effectively utilized as an objective ADL evaluation tool that helps doctors diagnose dementia based on ADL data of senior citizens who live alone in smart home sections in actual residential areas.

Second, this study reflected a difference of individual daily patterns. Specifically, the personalized model reflected differentiated anomaly detection standards based on individual cognitive abilities. A random forest (RF) algorithm was used to compare the accuracy of the personalized model and that of the non-personalized model. The comparison result indicated that the accuracy (91.20%) of the personalized model was higher than that (84.54%) of the non-personalized model, thereby verifying the excellent performance of the personalized model. The results showed that the life patterns of using household appliances during the late-night hours (00:00–05:00) were identified as an important feature in classifying normal controls and early-stage dementia patients.

Finally, the results of this study confirmed that the IoT big data-based normal controls–early-stage dementia auto-classification model demonstrated superior performance to the ADL-based model. The significance of this finding is that superior performance was derived when big data from the IoT sensors that reflect all aspects of daily living were used as features compared to the ADL features with limitations. That is, this study confirmed that normal controls–early-stage dementia auto-classification was possible without resorting to domain knowledge, indicating the major significance of this study for the objective diagnosis of dementia.

During this clinical study, home visits were difficult due to special circumstances of the COVID-19 pandemic, and it was also difficult to recruit participants for the clinical study; consequently, the numbers that participated in the clinical study were small and the study period was relatively short (13 months).

In this study, personalization was designed based on cognitive ability information obtained from the MMSE scores. Among ongoing research programs, the ‘Incubation Period Model for MCI prediction’ study [46] based on hospital records is underway. In the future, we plan to conduct research on automating the personalization process by integrating it with such studies.

## Figures and Tables

**Table 1:** Installation of sensors using ADL assessment items.

No	ADL Assessment Items	Place of Installation	Sensors Used
1	Cooking	Microwave oven	Door sensor
		Refrigerator	Vibration sensor
		Rice cooker	Vibration sensor
		Kitchen sink faucet	Vibration sensor
		Gas stove	Temperature–humidity sensor
		Kitchen	Motion sensor
2	Unlocking and closing entrance door	Entrance	Door sensor
		Household appliances	Smart plug
3	Using household appliances	Electric mat	Smart plug
		TV	Smart plug
		Fan	Vibration sensor
4	Household chores	Housecleaning—washing machine	Smart plug
		Housecleaning—bin, vacuum cleaner	Vibration sensor
		Washing dishes—Kitchen sink faucet	Vibration sensor
5	Grooming	Washbasin, showerhead—bathroom faucet	Vibration sensor
		Bathroom	Temperature–humidity sensor
6	Taking medications	Pill organizer	Vibration sensor
7	Indoor wandering	Path of indoor movement and gait speed	Lidar sensor
		Room	Motion sensor
		Living room	Motion sensor

**Table 2:** Example of IoT sensor data calculated in the time unit of one day.

Time	d1	d2	d3	m2	m3	m5	m6	p2	p3	p4	t1	v1	v2	v3	v4	v5
6 July 2020	11	4	2	90	3	22	10	5	0	0	1	1	7	0	2	4
7 July 2020	6	0	2	78	1	26	6	2	0	0	3	2	9	1	6	3
8 July 2020	6	2	0	111	3	9	8	4	0	0	1	4	8	0	0	2
10 July 2020	5	3	0	103	0	13	9	3	0	2	3	2	10	0	5	5
11 July 2020	13	4	0	115	0	36	9	4	0	2	0	0	10	0	4	2
13 July 2020	9	4	0	112	0	19	10	2	0	0	2	2	12	3	24	1
15 July 2020	7	5	1	125	0	23	9	3	0	0	3	1	11	0	10	5

Abbreviations: d: door sensor; m: motion sensor; p: smart plug; t: temperature–humidity sensor; v: vibration sensor.

**Table 3:** Personalization of ADL.

Category	Level of Cognitive Function	MMSE	Range of Personalized Anomaly Detection Criteria
Normal controls	No Cognitive Decline	30	When the MMSE score is out of “Lower Q1 − 1.5 × IQR/Upper Q3 + 1.5 × IQR”
	Very Mild Cognitive Decline	~	When the MMSE score is out of “Lower Q1 − 1.2 × IQR/Upper Q3 + 1.2 × IQR”
	Mild Cognitive Decline	24	When the MMSE score is out of “Lower Q1 − 1 × IQR/Upper Q3 + 1 × IQR”
Early-stage Dementia	Moderate Cognitive Decline	23 ~	When the MMSE score is out of “Lower Q1 − 0.5 × IQR/Upper Q3 + 0.5 × IQR”

**Table 4:** Type of analysis data.

Type of Analysis Data		Description
IoT sensor data	IoT Count	All counts detected by the vibration sensor or motion sensor
	IoT Duration	Duration of movement detected in front of the motion sensor
	Lidar	Indoor movement distance and gait speed detected by 2D-Lidar
ADL data	ADL Count	Number of times ADL activities were performed (6 types + indoor wandering)
	ADL Duration	Time taken for ADL activities (6 types + indoor wandering)

**Table 5:** ADL feature sets according to ADL categories.

ADL Item	Feature	Description
Indoor wandering	Movement in a room	Data of the participant’s movements in a room
	Indoor wandering late at night	All sensor data recorded between 00.00 and 5.00
Unlocking and closing entrance door	Going out	Data from the time of closing the door and to the opening of the door. In case there was a sensor that started operation, this case was not considered as going out.
	No locking of the entrance door	Data for cases of the sensor operation during the time of the participant’s going out.
Household chores	Laundering (washing machine)	Data of washing machine use from the start to the end of the washing machine operation
	Washing dishes	Data of using kitchen sink faucet for longer than 30 s
Cooking	Cooking	When two or more kitchen appliances had been used and the temperature of a gas stove had increased (including all cooking for less than 30 min)
	Breakfast cooking	Cooking between 5:00 and 10:00
	Lunch cooking	Cooking between 12:00 and 15:00
	Dinner cooking	Cooking between 17:00 and 20:00
	Cooking for over 30 min	Cooking data lasting longer than 30 min
	Cooking (gas stove—microwave oven)	When the sensors used during cooking included the gas stove and microwave oven
	Cooking (refrigerator—kitchen sink faucet)	When the sensors used during cooking included the refrigerator and kitchen sink faucet
	Cooking (refrigerator—gas stove)	When the sensors used during cooking included the refrigerator and gas stove
	Cooking (kitchen sink faucet— rice cooker)	When the sensors used during cooking included the kitchen sink faucet and rice cooker
	Heating food (microwave oven)	When the sensors used during cooking included the microwave oven but not the gas stove
Taking medications	Morning medications	Taking medications between 5:00 and 10:00
	Lunchtime medications	Taking medications between 12:00 and 15:00
	Evening medications	Taking medications between 17:00 and 20:00
	Medications before going to bed	Taking medications between 21:00 and 24:00
Grooming (personal hygiene)	Use of bathroom faucet (Nighttime)	All the data with the start time of the bathroom faucet use between 00:00 and 04:00
	Use of showerhead	Use of the showerhead installed in the bathroom faucet
	Use of bathroom faucet for more than 1 min	Use of bathroom faucet over 1 min but not the showerhead
	Bathroom faucet (total)	Data for all hours of bathroom faucet use
Using household appliances	TV (total)	Total hours of watching TV over 24 h
	TV watching in the morning	TV watching between 04:00 and 12:00
	TV watching at night	TV watching between 00:00 and 04:00
	TV after going out	Data of TV turned on for 30 min after the participant’s returning from going out
	Electric mat (total)	Total hours of using electric mat over 24 h
	Electric mat—Daytime	Use of electric mat between 12:00 and 16:00
	Electric mat—Nighttime	Use of electric mat between 00:00 and 04:00

**Table 6:** Characteristics of participants.

Category	Age	MMSE	CDR
Normal Controls (*n* = 7)	86	26	0
	79	29	0
	84	25	0
	72	27	0
	67	26	0
	74	30	0
	90	30	0
Average of Normal Controls	78.8	27.5	0
Early-stage dementia group (*n* = 6)	87	20	1
	76	13	1
	86	18	1
	76	30	0.5
	85	11	1
	72	14	1
Average of early-stage dementia group	80.3	17.6	0.92

**Table 7:** Analysis of statistical differences regarding use counts for the appliances used.

Sensor Location	*p*-Value
Entrance	0.03643 (<0.05)
Microwave oven	0.4745
Gas stove	6.431 × 10^−12^ (<0.05)
TV	4.363 × 10^−7^ (<0.05)
Washing machine	3.908 × 10^−7^ (<0.05)
Pill organizer	0.1878
Refrigerator	0.003404 (<0.05)
Rice cooker	0.03521 (<0.05)
Kitchen sink faucet	5.385 × 10^−7^ (<0.05)
Bathroom faucet	2.2 × 10^−16^ (<0.05)

**Table 8:** Analysis of differences in ADL duration.

ADL	*p*-Value
Grooming	6.728 × 10^−15^ (<0.05)
Using household appliances	1.063 × 10^−7^ (<0.05)
Cooking	0.01343 (<0.05)
Household chores	0.9674

**Table 9:** Differences in gait speeds between the normal controls and early-stage dementia groups.

Statistic	Gait Speed of Normal Controls (km/h)	Gait Speed of Early-Stage Dementia Group (km/h)
Min	0.600	0.6007
Median	0.930	1.0803
Mean	1.023	1.2091
Max	5.615	3.9251

**Table 10:** Status of training data.

Feature Set	Patients#	Data#	Mean Data Length	Feature# (IoT)	Feature#(ADL)
IoT	13 (NC: 7, Dem: 6)	20,184 (Dem: 7800, NC: 12,384)	1441 (Dem: 1114, NC: 1769)	132	.
ADL	13 (NC: 7, Dem: 6)	20,184 (Dem: 7800, NC: 12,384)	1441 (Dem: 1114, NC: 1769)	.	63
IoT + ADL	13 (NC: 7, Dem: 6)	20,184 (Dem: 7800, NC: 12,384)	1441 (Dem: 1114, NC: 1769)	132	63

**Table 11:** Model performance before and after application of personalization.

	Before Personalization			After Personalization		
	IoT	ADL	IoT + ADL	IoT	ADL	IoT + ADL
Precision	80.65%	63.99%	79.47%	85.29%	79.62%	88.47%
Recall	71.43%	59.08%	81.87%	82.62%	76.92%	90.03%
*F*1-*score*	75.76%	61.44%	80.65%	83.94%	78.25%	89.24%
Accuracy	80.98%	65.52%	84.54%	86.80%	83.47%	91.20%

**Table 12:** Major features of model applied with personalization.

No	Major Features with a Significant Impact
1	Duration of using electric mat (Late-night hours from 0:00 to 5:00 and Evening hours from 17:00 to 24:00)
2	Duration of using microwave oven (Late-night hours from 0:00 to 5:00)
3	Duration of TV watching (Late-night hours from 0:00 to 5:00)
4	Duration of using cooking appliances (Refrigerator-gas stove)
5	Duration of using gas stove (Daytime hours from 11:00 to 17:00 and Evening hours from 17:00 to 24:00)
6	Duration of using showerhead
7	Duration of using entrance
8	Duration of using bathroom faucet
9	Duration of using washing machine (Morning hours from 5:00 to 11:00)
10	Duration of using refrigerator
11	Duration of washing dishes
12	Duration of using cooking appliances (refrigerator-kitchen sink faucet)

## Data Availability

The data presented in this study are available on request from the corresponding author.
